# A validated prognostic nomogram for patients with H3 K27M-mutant diffuse midline glioma

**DOI:** 10.1038/s41598-023-37078-0

**Published:** 2023-06-20

**Authors:** Youheng Peng, Yanming Ren, Bowen Huang, Jun Tang, Yan Jv, Qing Mao, Yanhui Liu, Yinjie Lei, Yuekang Zhang

**Affiliations:** 1 Department of Neurosurgery, West China Hospital of Sichuan University, No. 37, Guoxue Alley, Chengdu, 610041 Sichuan People’s Republic of China; 2 College of Electronics and Information Engineering, Sichuan University, No. 24, South Section 1, First Ring Road, Chengdu, 610041 Sichuan People’s Republic of China

**Keywords:** CNS cancer, CNS cancer, Cancer in the nervous system

## Abstract

H3 K27M-mutant diffuse midline glioma (H3 K27M-mt DMG) is a rare, highly invasive tumor with a poor prognosis. The prognostic factors of H3 K27M-mt DMG have not been fully identified, and there is no clinical prediction model for it. This study aimed to develop and validate a prognostic model for predicting the probability of survival in patients with H3 K27M-mt DMG. Patients diagnosed with H3 K27M-mt DMG in the West China Hospital from January 2016 to August 2021 were included. Cox proportional hazard regression was used for survival assessment, with adjustment for known prognostic factors. The final model was established using the patient data of our center as the training cohort and data from other centers for external independent verification. One hundred and five patients were ultimately included in the training cohort, and 43 cases from another institution were used as the validation cohort. The factors influencing survival probability in the prediction model included age, preoperative KPS score, radiotherapy and Ki-67 expression level. The adjusted consistency indices of the Cox regression model in internal bootstrap validation at 6, 12, and 18 months were 0.776, 0.766, and 0.764, respectively. The calibration chart showed high consistency between the predicted and observed results. The discrimination in external verification was 0.785, and the calibration curve showed good calibration ability. We identified the risk factors that affect the prognosis of H3 K27M-mt DMG patients and then established and validated a diagnostic model for predicting the survival probability of these patients.

## Introduction

H3 K27M-mutant diffuse midline glioma (H3 K27M-mt DMG) was initially introduced as a new entity in the World Health Organization (WHO) classification of central nervous system tumors in 2016 and was recently renamed “diffuse midline glioma, H3 K27 altered” in the new 2021 WHO classification^1,2^. This is a kind of invasive tumor, which corresponds to WHO grade 4 with a considerably poor prognosis. The patients diagnosed with this tumor are mainly children. Nevertheless, there is no lack of adult patients^3^. Even after surgery and adjuvant radiotherapy and/or chemotherapy, the median survival is less than 1 year, the 2-year survival rate of patients is less than 10%, and the prognosis of children is worse than that of adults^4–6^.

Presently, there is only a preliminary understanding of the pathogenesis of H3 K27M-mt DMG. The methylation of the K27 residue on normal histone 3 can inhibit gene transcription. However, due to the missense mutation caused by the replacement of lysine with methionine at position 27 in histone variants HIST1H3B (H3.1) or H3F3A (H3.3), the methylation of histone 3 itself is reduced, which affects the stability of gene transcription^7–9^. This mutation leads to significant genetic, epigenetic, and clinical differences from wild-type DMG and other high-grade gliomas^7,10–12^. These differences also contribute to factors affecting the prognosis of patients with H3 K27M-mt DMG that may not be exactly the same as those of wild-type DMG^13,14^.

Some clinical factors are closely related to the prognosis of patients. At present, it is generally believed that age, Karnofsky Performance Scale (KPS) score and postoperative radiotherapy will affect prognosis^5,15,16^, while the influence of tumor location, tumor resection extent, chemotherapy, ATRX expression, p53 positivity, and Ki-67 index on prognosis is still controversial^16–20^; on the other hand, patient sex and histone 3 mutation type have no significant effect on prognosis^5^. However, due to the rarity of H3 K27M-mt DMG, only a few international reports exist and large cohort studies are very limited^3,21–23^. Therefore, the clinical factors affecting the prognosis of H3 K27M-mt DMG patients have not been fully explored. In terms of treatment, it is unclear whether the resection scope should be expanded and whether every patient should be recommended for postoperative radiotherapy or chemotherapy^24–26^.

A nomogram is a kind of statistical model that can make a personalized prediction based on the characteristics of the patient using a linear scale. It can help neurooncologists accurately assess the risk of specific endpoints to individual patients and help clinicians make personalized treatment decisions. At present, many survival prediction models for high-grade and low-grade gliomas have been developed^27–30^. Nevertheless, to our knowledge, there is no clinical prediction model for patients with H3 K27M-mt DMG. The purpose of our study was to further explore the factors affecting the prognosis of H3 K27M-mt DMG patients based on data obtained from our institution and to develop and validate a prognostic prediction model based on clinical manifestations, molecular pathology, and other factors to accurately predict the individualized survival probability of postoperative H3 K27M-mt DMG patients and help clinicians make clinical decisions.

## Methods

### Study design and patient cohort

For the training dataset, we reviewed cases of H3 K27M-mt DMG diagnosed and treated in the West China Hospital from January 2016 to August 2021. All patients underwent surgical resection or stereotactic biopsy and were confirmed to have H3 K27M-mt DMG. Except for a child under 3 years old, all patients are suggested to receive radiotherapy and chemotherapy. Radiotherapy was performed after surgery with a dose of 45–60 Gy, which was divided into 30 daily fractions. During the radiotherapy period, concomitant temozolomide based chemotherapy was administrated at a dose of 75 mg/m^2^/d. One month after the completion of radiotherapy, an adjuvant chemotherapy regimen was initiated. The major chemotherapy regimen was the Stupp protocol: 150–200 mg/m^2^ TMZ daily for 5 consecutive days, with 6 cycles given every 4 weeks, if tolerated.

**Figure 1:**
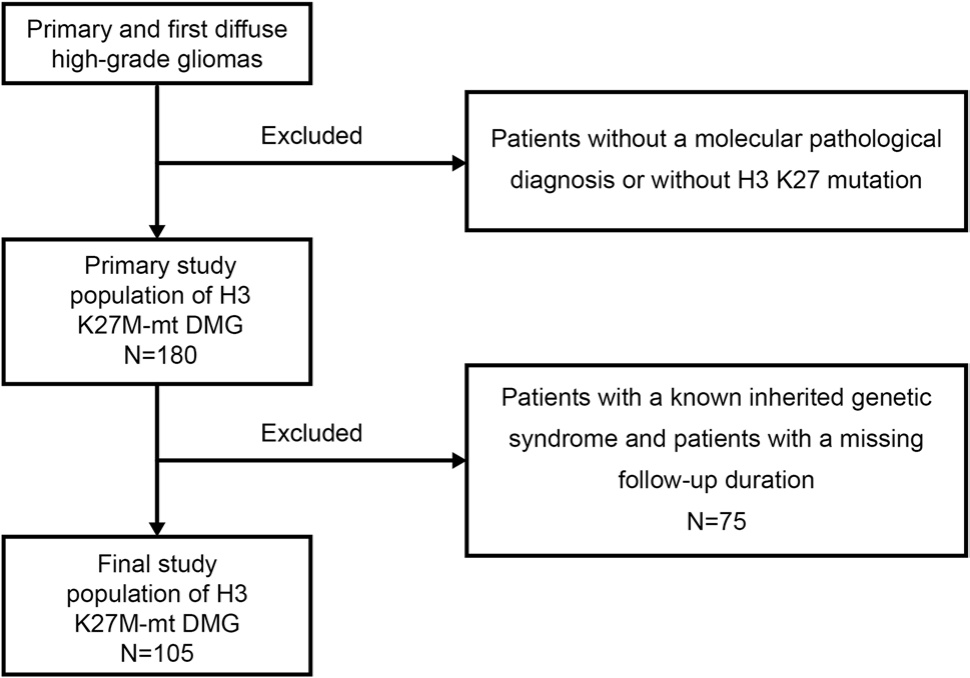
Flowchart of patient selection for the training dataset.

The relevant demographic and clinical data on all glioma cases were obtained and identified from the institutional Hospital Information System (HIS). After excluding patients with a known inherited genetic syndrome and patients with missing follow-up duration, a total of 105 cases were included in the training set (Fig. 1). The detailed information of patients in the training set can be found in Supplementary Table 1. According to the inclusion and exclusion criteria, 43 cases from another institution were used as the validation cohort (Supplementary Table 2)^15^. The original authors of the external dataset has declared that their data can be got without undue reservation. Based on existing data and previous literature reports, combined with clinical experience, relevant outcome predictors were obtained from each patient: survival/follow-up time in months, survival status (dead or alive), age at diagnosis (continuous), sex (male or female), preop KPS score (20–100), tumor location (supratentorial or infratentorial), extent of resection (GTR, STR, PR or biopsy), adjuvant therapy (RT or TMZ), ATRX expression (lost or intact), p53 positivity (positive or negative), Ki-67 level (continuous), and *MGMT* status (unmethylated or methylated). The study was conducted according to the guidelines of the Declaration of Helsinki, and approved by the West China Hospital of Sichuan University Biomedical Research Ethics Committee (protocol code: 1186). Written informed consent was obtained from each patient or the patient’s legal guardian.

### Definitions

Tumor location was determined by magnetic resonance imaging (MRI) reviewed by two neurosurgeons. The primary site of the tumor was divided into supratentorial and infratentorial. Regarding the degree of resection, gross total resection (GTR) was defined as no tumor residue (100%), subtotal resection (STR) was defined as more than 90% of the resection range, and partial resection (PR) was defined as less than 90% but more than 50% of the tumor resected.

The histopathological results were reviewed by the neuropathologist according to the WHO classification of central nervous system tumors in 2016. For ATRX, loss of nuclear staining was assessed. For *p53*, strongly positive staining in > 30% of tumor cell nuclei was considered *p53* positive. Methylation-specific quantitative PCR was used to investigate the status of O6-methylguanine DNA methyltransferase (*MGMT*) promoter methylation. Real-time quantitative PCR was performed at 95 °C for 5 min, followed by 45 cycles at 95 °C for 20 s, 56 °C for 30 s, and 72 °C for 20 s. The *MGMT* methylation status was considered positive when the Ct value was < 28.

The polymerase chain reaction (PCR) primers for *H3F3A, HIST1H3B* were designed^21,31^. Standard buffer conditions, 50 ng of DNA, and Taq DNA polymerase (TaKaRa, China) were used. The reaction mixture was subjected to an initial denaturation at 95 °C for 10 min, followed by 35 cycles of amplification (denaturation at 95 °C for 30 s, annealing at 56 °C for 15 s, and extension at 72 °C for 20 s). PCR products were sequenced in both the sense and antisense directions by Sangon Biotech (Chengdu, China).

### Statistical analyses

Descriptive statistics were used to analyze the frequency of participant characteristics. Categorical variables were expressed as percentages and compared using the chi-square test. Significant differences between the two groups were analyzed using Student’s t test (2-tailed), and the Wilcoxon rank-sum test was used for Ki-67 expression level and follow-up time. Overall survival (OS) was defined as the time from diagnosis to death; OS was examined for each dataset using the Kaplan–Meier method and was compared between the two datasets using the log-rank test.

The correlation between each factor and OS was assessed by the univariate Cox proportional hazards model. At the same time, the factors with a significant impact on prognosis were further screened through the L1-pennalized (Lasso) regression model. Factors with a statistically significant hazard ratio (*p* < 0.05) were selected in the multivariable Cox proportional risk model. The proportional risk assumption of all analyzed variables was confirmed. Finally, based on the multivariable Cox proportional hazards model, a prognostic model based on a nomogram was established.

Internal validation used bootstrap resampling to evaluate the stability of the prediction model. The validation was performed in 1000 boot iterations extracted from the development samples. The model was rebuilt in each bootstrap iteration and tested on the original sample to evaluate its performance. External validation was performed with the validation set. The prediction model was evaluated in the external verification using the concordance index (c-index) and calibration curve. The c-index refers to the proportion of all patient pairs whose predicted results are consistent with the actual results and estimates the probability that the expected results are consistent with the actual observed results. Its value range is 0.5–1. The closer the index is to 1, the better the discrimination of the model. Calibration of the final model was established as follows: all patients were assigned into quartiles of the nomogram-predicted 6-, 12-, and 18-month survival probabilities, and within each quartile, the mean nomogram-predicted 6-, 12-, and 18-month survival probability was plotted against the Kaplan‒Meier-estimated 6-, 12-, and 18-month survival. The diagonal of the calibration curve indicates complete calibration, and the relative deviation above or below this line indicates the error estimation of the survival probability; it can be visually assessed.

All statistical analyses were performed in IBM SPSS Statistics version 22 (IBM Corp.) and R software version 4.2.0 (R Foundation for Statistical Computing; http://www.R-project.org, 2017). The following R packages were used: RMS package (version 6.3-0) for the nomograms and calibration curves, glmnet package (version 4.1-4) for the Lasso regression model, survminer package (version 0.4.9) for plotting of Kaplan–Meier curves, and pec package (version 2022.05.04) for bootstrap resampling and c-index calculation.

**Figure 2:**
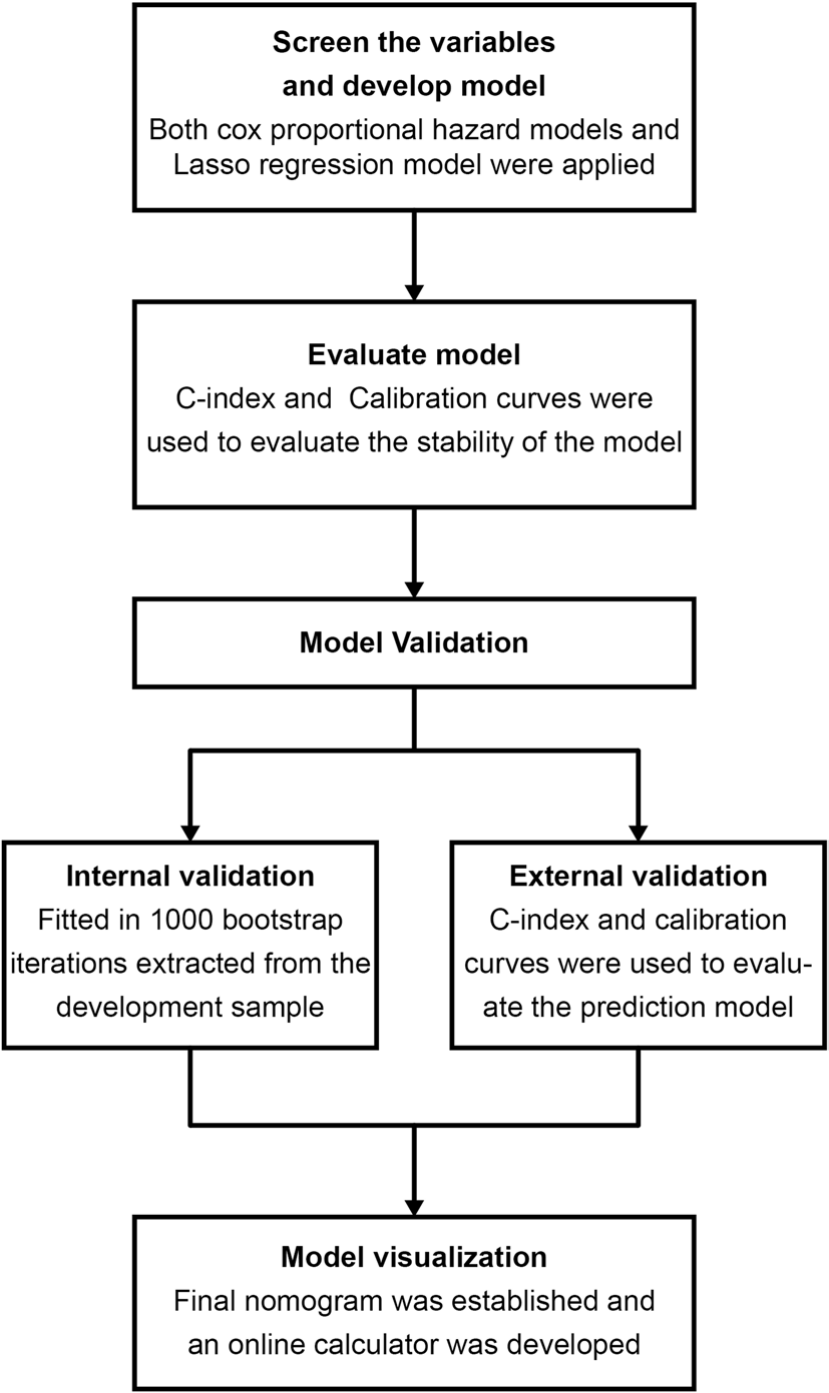
Flowchart of statistical analyses.

Figure 2 shows the statistical analyses in entirety.

## Results

### Demographic and clinical features

A total of 105 patients were included in the training cohort. The validation cohort included 43 patients. The mean age at the time of diagnosis in the training cohort (23.88 ± 17.059 years) was significantly lower than that in the validation cohort (35.4 ± 19.388 years, *p* < 0.001). In the training cohort, the number of male and female patients was similar (male 48.57%, female 51.43%), while in the validation cohort, there were more male patients than female patients (male 60.47%, female 39.53%). There was no significant difference in sex distribution, preoperative KPS score distribution, or tumor location between the two cohorts (*p* = 0.189, *p* = 0.264, *p* = 0.704). The number of patients receiving radiotherapy in the training cohort (20.95%) was significantly lower than that in the validation cohort (55.81%, *p* < 0.001), as was the distribution of patients receiving temozolomide chemotherapy (25.71% in the training group and 53.49% in the validation group, *p* < 0.001).

There were significant differences in the distribution of ATRX expression and p53 positivity between the two cohorts (*p* < 0.001). The ATRX intact rate (70.48%) in the training group was significantly higher than that in the validation group (23.26%), and the positivity rate of p53 in the training group (72.38%) was also considerably higher than that in the validation group (53.49%). There was no significant difference in the distribution of *MGMT* promoter methylation and Ki-67 expression between the two cohorts (*p* = 0.477, *p* = 0.503). The average follow-up time was 9.68 months in the training cohort and 11.94 months in the validation cohort. In univariate analysis, the overall survival time of patients in the validation cohort was significantly longer than that in the training cohort (*p* = 0.04).

Table 1 summarizes the baseline characteristics of patient variables in both datasets.

**Table 1:** Baseline characteristics of H3 K27M-mt DMG in the training and validation cohorts.

Variable	Training cohort n = 105	Validation cohort n = 43	*p* value
Gender, n (%)			0.189**
Male	51 (48.57)	26 (60.47)	
Female	54 (51.43)	17 (39.53)	
Mean age at diagnosis, years	23.88 (± 17.059)	35.4 (± 19.388)	< 0.001*
Children	53 (53.48)	10 (23.26)	0.002**
Adult	52 (49.52)	33 (76.74)	
Tumor location, n (%)			0.704**
Supratentorial	55 (52.38)	24 (55.81)	
Infratentorial	50 (47.62)	19 (44.19)	
Extent of resection, n(%)			0.95**
GTR	20 (19.05)	8 (18.60)	
STR	26 (24.76)	12 (27.91)	
PR	51 (48.57)	19 (44.19)	
Biopsy	8 (7.62)	4 (9.30)	
Pre-op KPS, n (%)			0.264**
20	2 (1.90)	1 (2.33)	
30	4 (3.81)	5 (11.63)	
40	3 (2.86)	1 (2.33)	
50	4 (3.81)	1 (2.33)	
60	10 (9.52)	5 (11.63)	
70	24 (22.86)	8 (18.60)	
80	38 (36.19)	8 (18.60)	
90	19 (18.10)	14 (32.56)	
100	1 (0.95)	0 (0.00)	
Radiotherapy, n (%)			< 0.001**
Yes	22 (20.95)	24 (55.81)	
No	83 (79.05)	19 (44.19)	
TMZ chemotherapy, n (%)			0.001**
Yes	27 (25.71)	23 (53.49)	
No	78 (74.29)	20 (46.51)	
ATRX expression, n (%)			< 0.001**
Intact	74 (70.48)	10 (23.26)	
Lost	21 (20.00)	33 (76.74)	
Missing	10 (9.52)	0 (0.00)	
p53 positivity, n (%)			< 0.001**
Positive	76 (72.38)	23 (53.49)	
Negative	13 (12.38)	20 (46.51)	
Missing	16 (15.24)	0 (0.00)	
Ki67 expression (range)	0.2324 (0.02–0.70)	0.2398 (0.01–0.70)	0.503***
MGMT promoter methylation, n (%)			0.817**
Methylated	18 (17.14)	10 (23.26)	
Unmethylated	58 (55.24)	29 (67.44)	
Missing	29 (27.62)	4 (9.30)	
Overall survival vital status, n (%)			< 0.001**
Dead	94 (89.52)	23 (53.49)	
Survive	11 (10.48)	20 (46.51)	
Mean follow-up, months (range)	9.68 (0.23–56.23)	11.94 (0.03–41.87)	0.04***

* *p* value calculated using the t-test.

** *p* value calculated using the chi-square test.

*** *p* value calculated using the Wilcoxon rank-sum test.

### Prognosis analyses and internal validation

**Figure 3:**
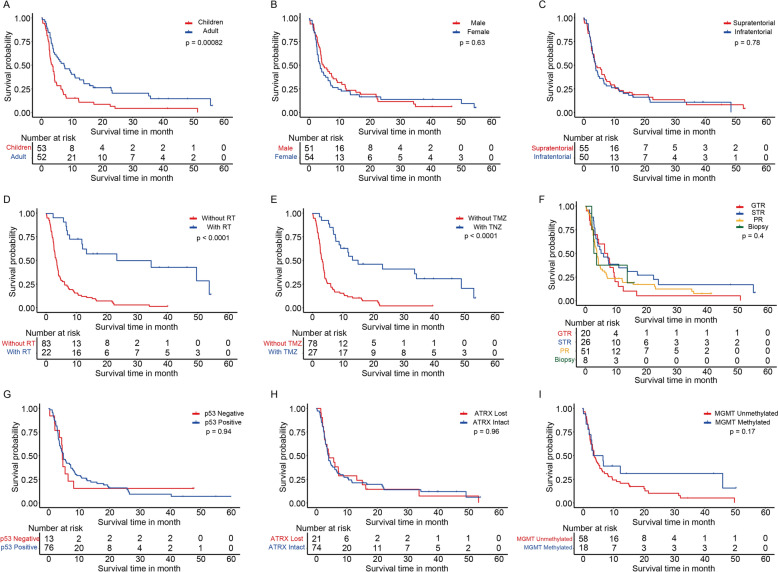
Kaplan–Meier curves for patients with H3 K27M-mt DMG by different variates. (**A**) Age group, (**B**) sex, (**C**) tumor location, (**D**) radiotherapy, (**E**) TMZ chemotherapy, (**F**) extent of resection, (**G**) p53 positivity, (**H**) ATRX expression, (**I**) MGMT promoter methylation.

As shown in Table 2, the univariate Cox proportional hazards model in the training cohort shows that H3 K27M-mt DMG OS is significantly correlated with several predictive factors, including age at diagnosis (children or adults), preop KPS score (20–100, in increments of 10), radiotherapy (RT) (yes or no), temozolomide chemotherapy (TMZ) (yes or no), and Ki-67 expression (continuous variable). Kaplan–Meier curves were plotted to compare the effects on H3 K27M-mt DMG patients by different variables. The results showed significant differences according to age group (*p* = 0.00082) and treatment status with radiotherapy (*p* < 0.0001) or TMZ chemotherapy (*p* < 0.0001) (Fig. 3).

**Table 2:** Uni- and multivariable Cox proportional hazards analysis for H3 K27M-mt DMG in the training and validation cohorts.

Predictor	Univariate Hazard Ratio	95% CI	*p* value	Multivariate Hazard Ratio	95% CI	*p* value
Gender (male vs. female)	1.106	0.734–1.667	0.628			
Age (≥ 16 years vs. < 16 years)	0.498	0.329–0.755	**0.001**	0.5621	0.363–0.870	0.010
Anatomic location (Infratentorial vs. Supratentorial)	1.062	0.707–1.596	0.773			
Extent of resection						
GTR versus Biopsy	1.1019	0.441–2.755	0.836			
STR versus Biopsy	1.1481	0.488–2.701	0.752			
PR versus Biopsy	0.7385	0.296–1.842	0.516			
Pre-op KPS	0.964	0.952–0.976	**1.07e−08**	0.959	0.947–0.971	1.54e-10
Radiotherapy (yes vs. no)	0.186	0.097–0.356	**4.21e−07**	0.250	0.103–0.603	0.002
TMZ chemotherapy (yes vs. no)	0.235	0.135–0.408	**2.82e−07**	0.5378	0.270–1.072	0.078
ATRX expression (lost vs. intact)	0.986	0.596–1.633	0.957			
p53 positivity (positive vs. negative)	0.979	0.515–1.860	0.947			
Ki-67 expression	8.966	2.276–35.330	**0.002**	6.4656	1.437–29.086	0.015
MGMT (methylated vs. unmethylated)	0.649	0.351–1.199	0.168			

*GTR* gross total resection, *STR* subtotal resection, *PR* partial resection, *TMZ* temozolomide, *KPS* Karnofsky performance status, *MGMT* O6-methylguanine-DNA methyltransferase

Significant values are in [bold].

**Figure 4:**
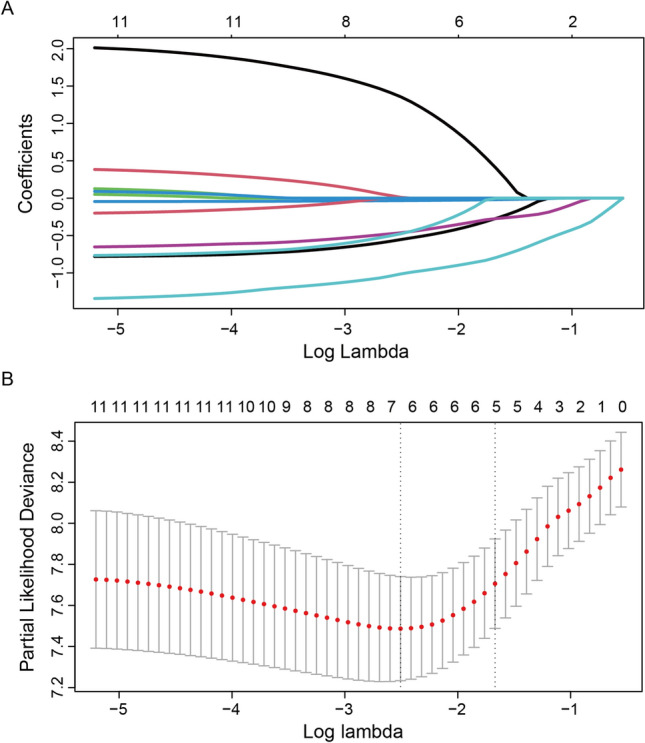
L1-penalized (Lasso) regression models were applied to further identify prognostic factors in the training cohort. According to the minimum criterion (left dotted line), log (lambda), − 2.52, Age, preoperative KPS, RT, TMZ, MGMT, and Ki-67 expression were determined to be significant (**A**). LASSO coefficient profiles of the features. The left vertical dotted line indicates the optimal value based on the minimum criterion giving 6 non-zero coefficients (**B**).

In the final multivariable Cox proportional hazards model, the four predictors were still significant, and the TMZ factor was at the critical value (*p* = 0.078). Meanwhile, the Lasso regression model was also applied to further identify prognostic factors for the OS of H3 K27M-mt DMG patients (Fig. 4)^32^. The final predictors included in the model construction were age at diagnosis, preoperative KPS, RT and Ki-67 expression.

**Figure 5:**
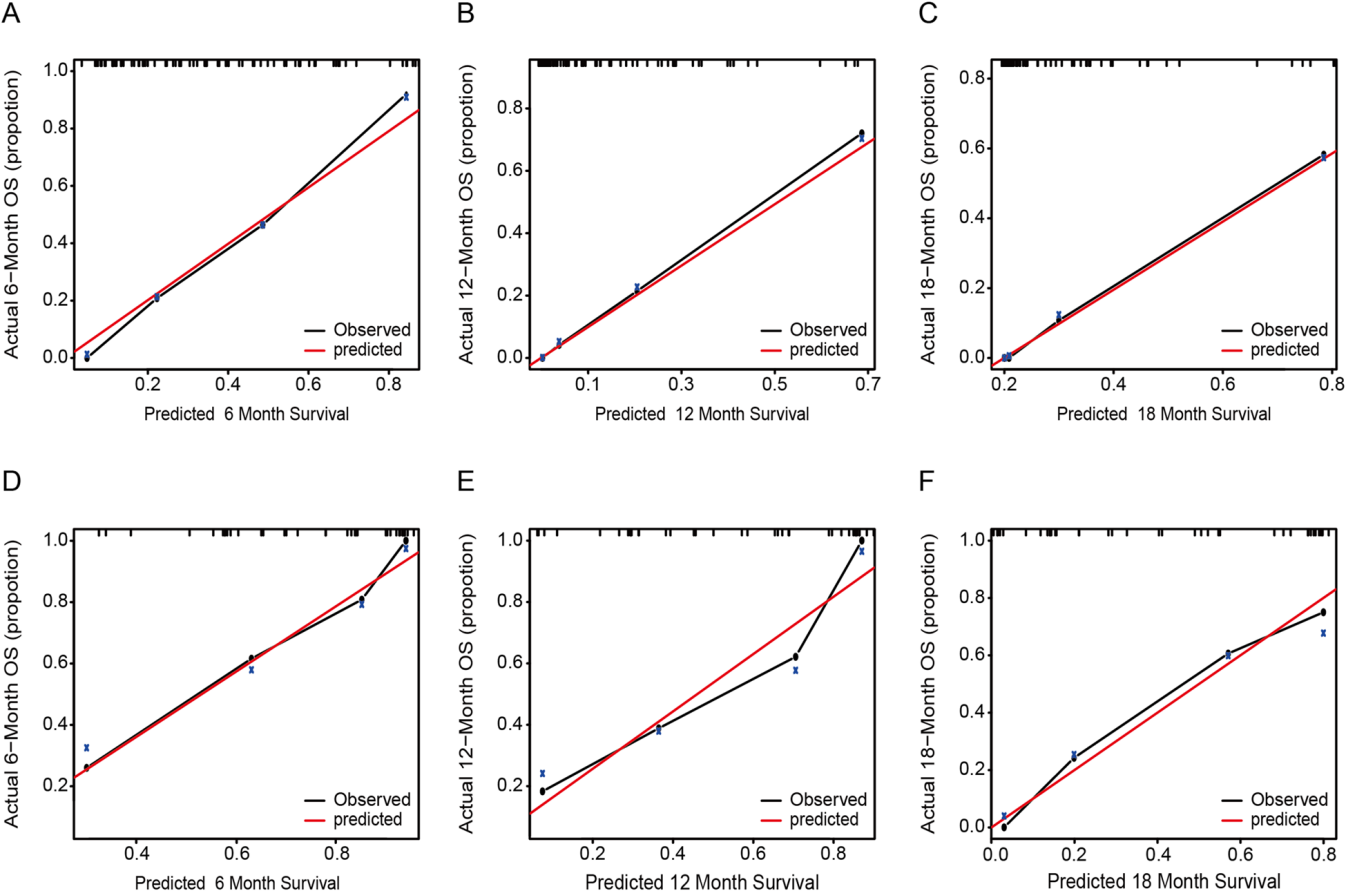
(**A**–**F**) Calibration curves for the nomogram training data at 6 months (**A**), 12 months (**B**), and 18 months (**C**) and independent validation data at 6 months (**D**), 12 months (**E**), and 18 months (**F**).

Bootstrap internal validation showed that the model was not overfitting-corrected. The corrected c-index was 0.776, 0.766, and 0.764 at 6, 12, and 18 months, respectively. All corrected c-indices exceeded 0.75 at three times, indicating that our model has relatively good discrimination. The black line in the calibration curves represents the observed survival rates, and the gray line represents the ideal survival rates. Although the 12-month and 18-month survival probability is slightly higher than the diagonal, indicating that the 12-month and 18-month survival rates predicted by the nomogram are lower than those observed, in general, the two lines are closely aligned, showing excellent calibration between the predicted and observed data (Fig. 5).

### Assessment and validation of the model

**Figure 6:**
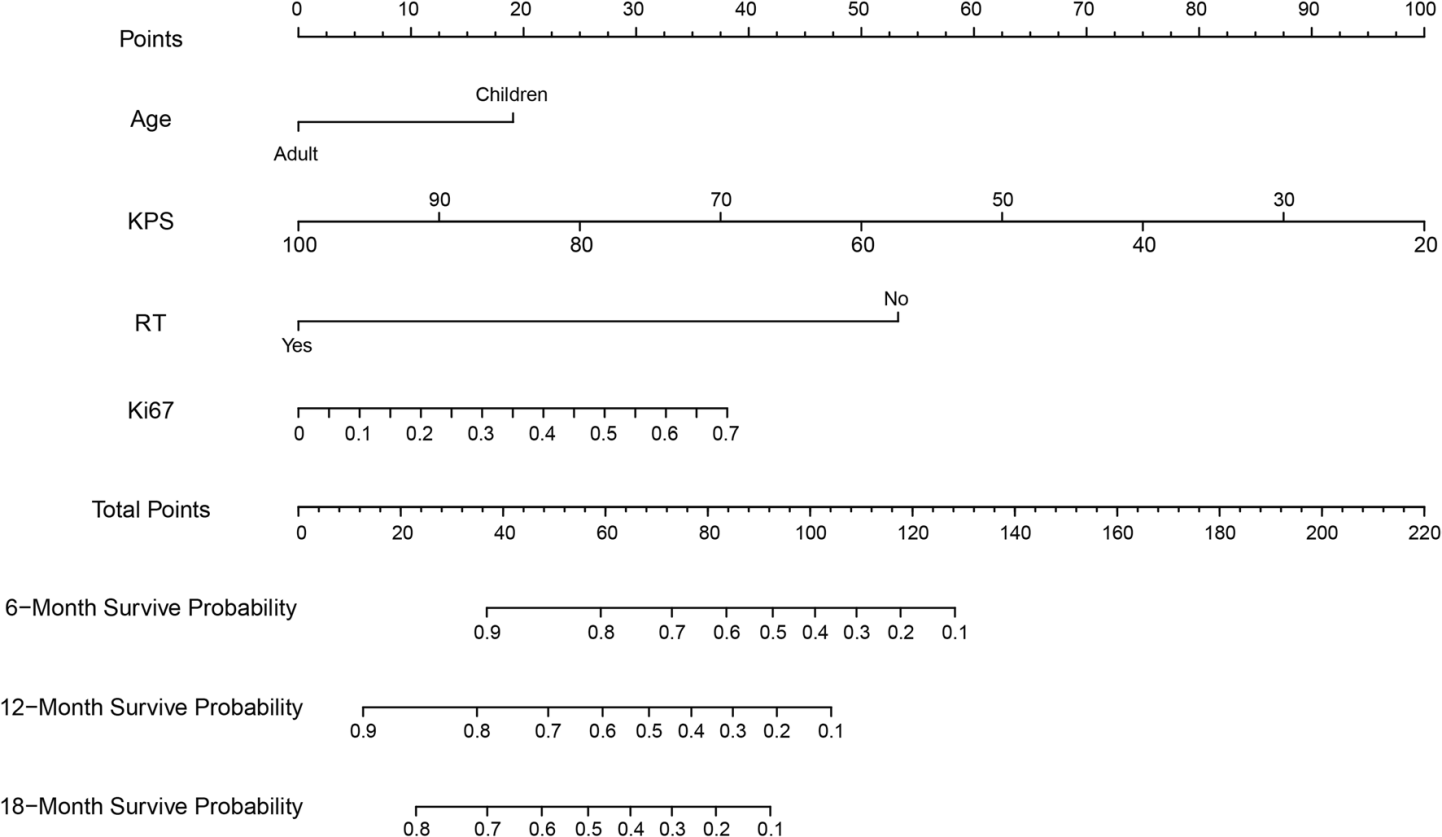
Nomogram to predict the survival rate of patients with H3 K27M-mt DMG at 6, 12, and 18 months.

Finally, we used the training set to construct a nomogram to estimate its 6-month, 12-month, and 18-month survival probability and performed independent verification on the external dataset (Fig. 6).

In external verification, the model performed well in discrimination and correction (Fig. 5). The c-index of the validation set was 0.785. For each quartile group, the estimated versus observed 6-month, 12-month, and 18-month survival probabilities intersected the diagonal, indicating that the predicted value approximated the observed value within a 95% confidence interval.

## Discussion

To the best of our knowledge, this study is the first prognostic prediction model developed for H3 K27M-mt DMG. The development and improvement of the previous glioma prognosis prediction model may not be suitable to apply H3 K27M-mt DMG because the clinical factors affecting the prognosis between them are not completely consistent. Therefore, developing a prognostic prediction model for H3 K27M-mt DMG is necessary. We developed a practical H3 K27M-mt DMG prognosis prediction model using data from our center and independently verified it with external datasets. Cox proportional hazards regression analysis was used in the prediction model. The model included the following variables: age at diagnosis, preoperative KPS score, receipt of radiotherapy and Ki-67 level. The training cohort used to build the model contains comprehensive clinical information (such as tumor location, resection range, ATRX expression, and *MGMT* promoter methylation), which helped to create a robust model. The prediction model performs well in internal and external validation. The predictive factors in the model are closely related to the prognosis and are easy to measure and obtain.

We did not select *IDH1* and *IDH2* as factors that may affect the prognosis. As reported in other studies, no *IDH1* mutation was found in our cohort. The same conclusion was reached on pathological molecular markers such as GFAP and TERT^22,33,34^. In the cohort from our center, the H3 K27M-mt DMG mutation subtype is mostly the *H3.3* mutation. There is little *H3.1* mutation, so we did not take the mutation subtype as a factor in the Cox proportional hazards model. Although it has been reported that the mutation subtype has no significant impact on prognosis^5^, the H3 mutation subtype should be included in future work to explore its effect on prognosis and whether there are changes in the risk ratio of other factors affecting prognosis under different H3 mutation subtypes. Considering the convenience of clinical practice, we divided the age at the time of diagnosis into child (≤ 16) and adult groups (> 16). In terms of treatment, for the management of general gliomas, surgery is the basis of diagnosis and treatment^35^. Despite the lack of randomized controlled trials of surgery, there is increasing evidence that extensive tumor resection can have a survival advantage in the treatment of all grades of gliomas^36^. However, in our cohort, the effect of tumor resection extent on prognosis was not significant, which may also be related to the location of the tumor and different oncological behaviors of H3 K27M-mt DMG^21^. In terms of adjuvant therapy, the effect of radiotherapy on the prognosis of H3 K27M-mt DMG is more accurate, but TMZ effects had not yet been determined. This may be due to the large unmethylated *MGMT* gene promoter present in some patients, resulting in temozolomide resistance in H3 K27M-mt DMG, or the fact that patients with severe conditions sometimes take more active chemotherapy^37^. The univariate Cox proportional hazards regression analysis and Lasso regression model showed that TMZ was a significant prognostic factor in our training cohort, but it showed no statistical significance in multivariate Cox proportional hazard regression analysis, so we didn’t include it in the model ultimately.

### Strengths and limitations

This study has strengths. The sample size was more than 100 H3 K27M-mt DMG cases, and thus, to our knowledge, the study included one of the largest case groups among existing studies. In addition to the univariate Cox proportional hazards model and multivariate Cox proportional hazards model, we also used the Lasso regression model to screen variables, which makes the model we built with the training dataset more stable. In addition, we used independent external data for verification, which proved the universality of our model, avoided overfitting of the model, and showed the application value of our model. Finally, we provided a nomograph based on the model and developed a web calculator for it, which makes it easier for clinicians to use in clinical work.

This study also has limitations. First, the baseline characteristics of the development and validation datasets were significantly different (Table 1), and the amount of data used for external validation was not large enough. However, even with these problems, the model still demonstrates a good calibration effect, showing the model's reliability. Second, although we have recommended that all patients receive radiotherapy and TMZ chemotherapy, it is frustrating that the compliance of our patients is not ideal, resulting in a low number of cases that eventually receive radiotherapy and TMZ chemotherapy (30/105, 28.57%). More patients who received TMZ chemotherapy need to be included in future work to explore the specific impact of TMZ on prognosis. These may become independent prognostic factors to help build more effective prognostic models. We will also discuss these predictive factors in future work.

## Conclusion

This study established and validated a prognostic model for predicting the survival probability of patients with H3 K27M-mt DMG. The model has the characteristics of being easy to use, fast, and easily available with strong interpretability and generalization ability and high discrimination and calibration performance. We have established a simple and easy-to-use nomogram based on the Cox proportional hazards model for clinical application. To facilitate the clinical use of this nomogram, free software for its implementation is provided (http://120.48.173.14:5000).

## Supplementary Information

Supplementary Table 1.

Supplementary Table 2.

## Data Availability

All data generated or analysed during this study are included in the supplementary tables.
